# A Critical Investigation of Cerebellar Associative Learning in Isolated Dystonia

**DOI:** 10.1002/mds.28967

**Published:** 2022-03-21

**Authors:** Anna Sadnicka, Lorenzo Rocchi, Anna Latorre, Elena Antelmi, James Teo, Isabel Pareés, Britt S. Hoffland, Kristian Brock, Katja Kornysheva, Mark J. Edwards, Kailash P. Bhatia, John C. Rothwell

**Affiliations:** ^1^ Department of Clinical and Movement Neurosciences University College London London UK; ^2^ Movement Disorders and Neuromodulation Group St. George's University of London London UK; ^3^ Department of Medical Sciences and Public Health University of Cagliari Cagliari Italy; ^4^ Department of Neuroscience, Biomedicine and Movement University of Verona Verona Italy; ^5^ Department of Neurosciences Kings College Hospital NHS Foundation Trust London UK; ^6^ Movement Disorders Program, Neurology Department Hospital Ruber Internacional Madrid Spain; ^7^ Department of Neurology Donders Institute for Brain, Cognition and Behaviour, Radboud University Medical Center Nijmegen the Netherlands; ^8^ Cancer Research UK Clinical Trials Unit University of Birmingham Birmingham UK; ^9^ School of Psychology University of Birmingham Birmingham UK

**Keywords:** dystonia, eyeblink conditioning, cerebellum, associative learning

## Abstract

**Background:**

Impaired eyeblink conditioning is often cited as evidence for cerebellar dysfunction in isolated dystonia yet the results from individual studies are conflicting and underpowered.

**Objective:**

To systematically examine the influence of dystonia, dystonia subtype, and clinical features over eyeblink conditioning within a statistical model which controlled for the covariates age and sex.

**Methods:**

Original neurophysiological data from all published studies (until 2019) were shared and compared to an age‐ and sex‐matched control group. Two raters blinded to participant identity rescored all recordings (6732 trials). After higher inter‐rater agreement was confirmed, mean conditioning per block across raters was entered into a mixed repetitive measures model.

**Results:**

Isolated dystonia (*P* = 0.517) and the subtypes of isolated dystonia (cervical dystonia, DYT‐TOR1A, DYT‐THAP1, and focal hand dystonia) had similar levels of eyeblink conditioning relative to controls. The presence of tremor did not significantly influence levels of eyeblink conditioning. A large range of eyeblink conditioning behavior was seen in both health and dystonia and sample size estimates are provided for future studies.

**Conclusions:**

The similarity of eyeblink conditioning behavior in dystonia and controls is against a global cerebellar learning deficit in isolated dystonia. Precise mechanisms for how the cerebellum interplays mechanistically with other key neuroanatomical nodes within the dystonic network remains an open research question. © 2022 The Authors. *Movement Disorders* published by Wiley Periodicals LLC on behalf of International Parkinson Movement Disorder Society.

Dystonia is a hyperkinetic movement disorder characterized by involuntary sustained muscle contractions which lead to twisting and repetitive movements or abnormal postures.^1^ Traditionally considered a disorder of basal ganglia function, recent research has pointed to abnormalities of multiple brain regions and within this wider sensorimotor network the cerebellum is thought to be a key node.^2^, ^3^, ^4^

Eyeblink conditioning is a cerebellar‐dependent experimental paradigm that has been traditionally used to study cerebellar function. Pavlovian by design, a biologically potent stimulus (the unconditioned stimulus) is paired with a previously neutral stimulus (the conditioning stimulus). Experimentally in humans the unconditioned stimulus is typically supraorbital nerve stimulation causing a blink (the unconditioned response). The conditioning stimulus (usually an auditory tone) occurs shortly before the unconditioned stimulus and with time the tone alone yields a conditioned blink (Fig. 1a). Informatively, eyeblink conditioning can be adapted and tested across species and elements of the paradigm can be specifically mapped to the function of individual cells within the cerebellar circuitry.^5^ For example, the magnitude of conditioned eyeblink responses correlates on a trial‐by‐trial manner to the level of firing of Purkinje cells (magnitude of simple spike suppression).^5^, ^6^ Eyeblink conditioning is therefore an attractive experimental method with the potential to map behavioral outcomes to the cerebellar micro‐circuitry.

**FIG 1:**
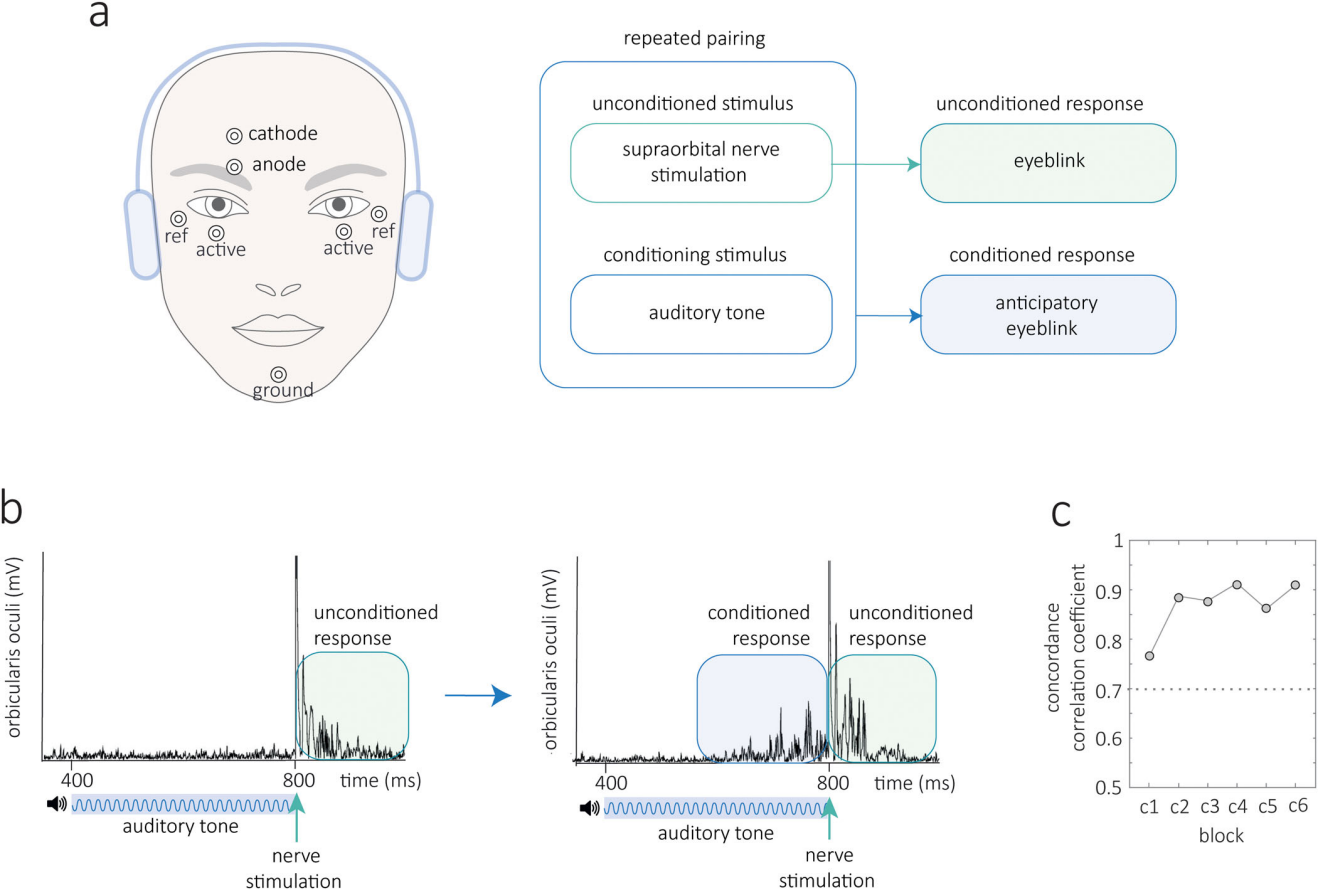
Eyeblink conditioning in humans. Method (**a**) the unconditioned stimulus consists of electrical stimulation to the supraorbital nerve at 800 ms which causes a blink, the unconditioned response. The conditioning stimulus is an auditory tone that starts at 400 ms with a duration of 400 ms. With repeated pairings a conditioned blink response emerges prior to supraorbital nerve stimulation. (**b**) Rectified electromyographic traces from a single trial early with no conditioned response and a later trial when conditioning has developed. (**c**) Two assessors scored the number of conditioned responses in every trial whilst blinded to the participants' identity. Their post hoc concordance correlation coefficient was excellent across all blocks (values >0.7 are considered reasonable, see supplementary methods for detail). [Color figure can be viewed at wileyonlinelibrary.com]

Impaired eyeblink conditioning is widely cited as evidence of functional cerebellar disturbance in the dystonia literature, yet, collectively findings across studies are conflicting. A preliminary study examined a mixed group of cervical dystonia and focal hand dystonia and found that both had lower levels of eyeblink conditioning.^7^ However, a later study documented normal eyeblink conditioning in cervical dystonia and reduced conditioning was only seen if there was coexisting head tremor.^8^ Eyeblink conditioning in genetic subtypes appear to be either normal in DYT‐*TOR1A‐*related dystonia or high in DYT‐*THAP1‐*related dystonia (unless an age‐matched control group is used).^9^ Thus low, normal, and high levels of eyeblink conditioning have been reported across subtypes of isolated dystonia. There are many potential reasons for the differences observed across studies. First, levels of eyeblink conditioning in the healthy population are highly variable. Clinical studies are therefore vulnerable to inconsistency if underpowered and uncontrolled covariates such as sex and age can confound.^10^, ^11^ Recent research also points to the subtypes of isolated dystonia having unique etiologies and/or neuroanatomical substrates and thus uniform patterns of eyeblink conditioning behavior across the subtypes are not necessarily anticipated.^12^

This current study capitalized on an unusual opportunity in isolated dystonia to collate raw data from individual studies. Our motivation was to examine whether isolated dystonia as a group is associated with altered eyeblink conditioning. We also evaluated whether there is any evidence that eyeblink conditioning is altered across subtypes of isolated dystonia and whether tremor or other clinical features influence conditioning. Clinical questions were probed within a statistical model that included age and sex as covariates, as young age and female sex are associated with higher levels of conditioning.^10^, ^11^

## 1 Methods

This collaborative project identified all studies that have examined eyeblink conditioning in isolated dystonia (until 2019). Original neurophysiological data were shared and a sex‐ and age‐matched control group were collected. Two raters blinded to participant identity scored all recordings (controls n = 50, dystonia n = 52, trials = 6732). After high inter‐rater agreement was confirmed (Fig. 1c), mean conditioning per block was entered into a mixed repetitive measures model to evaluate the influence of sex, age, dystonia, dystonia subtype, and clinical features. Full details of the methods are given in the supplementary information.

## 2 Results

**FIG 2:**
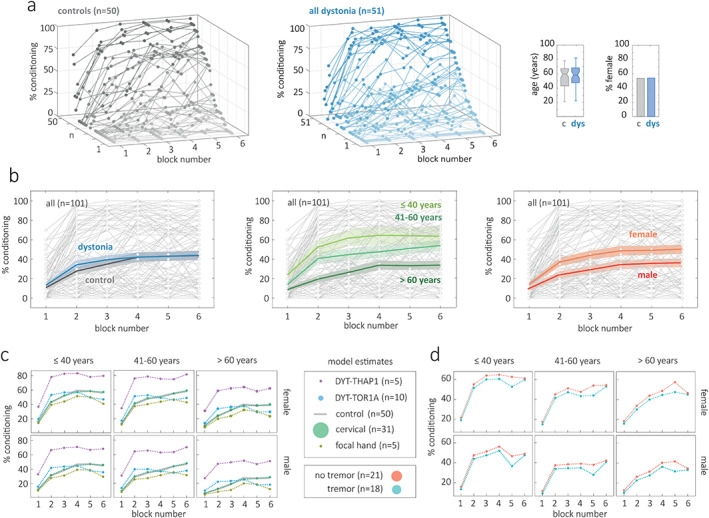
(**a**) The range of conditioning across individuals is demonstrated in three dimensional plots for controls (n = 50) and dystonia (n = 51). Block numbers 1 to 6 are shown on the *x*‐axis, percent (%) conditioning on the vertical *y*‐axis, and participant number on the *z*‐axis. For each group the profile of responses is sorted according to the percent conditioning achieved by the final block. Percent conditioning for each block is marked by a filled circle and connected by a thin line of the same color. The groups were matched for age and sex. (**b**) For each plot, all participants (n = 101) are plotted in pale gray in the background to illustrate degree of variability. Each graph then shows the group mean and shaded standard error when grouped by presence of dystonia, age range, and sex (control = grey, dystonia = blue, ≤40 years = light green, 41–60 years = green, >60 years = dark green, female = orange, male = red). (**c),** (**d**) On the *y*‐axis of each plot the model counterfactual is plotted, reflecting the model's best estimate for conditioning behavior by block for each age (horizontal panels) and sex category (vertical panels). In (**c**) controls are plotted with a partially transparent grey line to aid visualization of overlapping lines. The key shows the marker size proportional to the number of patients in each group. [Color figure can be viewed at wileyonlinelibrary.com]

A wide range of conditioning behavior was observed in both controls and patients with dystonia (Fig. 2a). Some subjects did not exhibit any conditioned responses (0% conditioning across all blocks) whereas others had conditioned responses counted in early blocks. The two groups had very similar demographic features in terms of age (t(99) = 0.0901, *P* = 0.928) and sex (equivalent proportions in each group). A plot of mean rates of conditioning by group (control, all dystonia) showed that eyeblink conditioning was similar to controls if all types of dystonia were considered as a group (Fig. 2b, *P* = 0.517). Younger age was associated with higher conditioning (Fig. 2b, *P* = 0.031). Higher levels of conditioning were also seen with female sex; a finding that was not significant in this study (*P* = 0.143), but has been observed reliably in previous studies.^11^

Model estimates for conditioning behavior for dystonia subtype across different age and sex categories are plotted in Figure 2c. Patients with DYT‐THAP1 dystonia had conditioning estimates 26 units higher than controls but there were only five patients in this in this subgroup (1 unit = 1% conditioning, *P* = 0.049). Otherwise, conditioning across subgroups was approximately equivalent with mean differences between dystonia subgroups and controls all <10 units (all *P* values >0.5).

Finally, we reviewed key clinical parameters to assess whether they influenced levels of conditioning. We examined disease features (severity, duration, tremor) and active treatments at the time of study (botulinum toxin injections and/or the medications trihexyphenidyl and clonazepam). The presence of tremor did not affect eyeblink conditioning in the ‘all’ dystonia group (Fig. 2d, *P* = 0.943) or the cervical dystonia subgroup (statistical comparison: *P* = 0.514). No other disease features or treatments at the time of study exhibited any clear effect over conditioning (all *P* values>0.5).

Based on the variance we observed in the final conditioning block we performed sample size calculations as a guide for future studies. The standard deviation in controls was 35.3%, standard deviation in dystonia 32.7%, with a mean standard deviation of 34.0%. Therefore, in order to detect a difference in conditioning of 5% a sample size of 739 per group is required. A difference of conditioning of 10% requires 185 per group, 20% requires 46 per group, and 30% requires 21 per group.

## 3 Discussion

Our results do not find evidence that isolated dystonia and the subtypes of isolated dystonia are associated with changes in eyeblink conditioning relative to controls. Our data suggest a revision to the idea that lower levels of eyeblink conditioning are a feature of focal dystonia.^7^ As eyeblink conditioning is often discussed as a proxy for cerebellar function, our findings have implications for how we define cerebellar involvement in dystonia pathophysiology.

Multiple convergent lines of evidence suggest that the cerebellum is involved in the pathophysiology of dystonia. For example, in humans cerebellar lesions can cause symptomatic/acquired dystonia, in genetic animal models cerebellar perturbations appear sufficient to generate dystonia and in focal dystonia imaging studies consistently point to cerebellar abnormalities.^13^, ^14^, ^15^ Therefore, there is collective evidence that the cerebellum is a key node within dystonic networks and cerebellar dysfunction may play a dominant role is some subtypes. However, we are still far from identifying specific cerebellar mechanisms. How do our results contribute to this discussion?

First, in general terms, our results suggest that there is a relative subtlety to any cerebellar dysfunction in genetic/idiopathic isolated dystonia. Neurodegenerative disorders such as spinocerebellar ataxia (type 6 and 8) affect the cerebellum relatively uniformly and are associated with broad functional impairments such as delayed eyeblink conditioning and impaired force field adaptation in reaching.^16^, ^17^ More circumscribed cerebellar deficits (eg, those caused by cerebellar stroke) can produce highly task‐specific impairments, such as abnormal adaptation to force but not to visuomotor adaptation and vice versa.^18^, ^19^ Force field and visuomotor adaptation paradigms have also been studied in isolated dystonia subtypes and there is not strong evidence that this movement calibration/computation is impaired.^20^, ^21^, ^22^, ^23^ Therefore, the absence of clear deficits in dystonia to these archetypal cerebellar paradigms and absence of overt cerebellar signs does suggest that there is a subtlety to cerebellar involvement.

To more precisely interpret our results, it is useful return to the observation that different elements of eyeblink conditioning have been mapped to the firing of individual cells within the cerebellar circuitry. As the cerebellar micro‐circuitry has a highly homogenous nature this has given rise to the idea that the cerebellum performs the same function at the algorithmic level across diverse domains (‘universal transform’).^24^ In such a scenario the broad functional heterogeneity of different cerebellar regions across motor control, perception, language, and cognition is primarily determined by connectivity patterns to cortical and subcortical targets rather than the micro‐circuitry.^24^ Therefore, if we interpret our results in line with the ‘universal cerebellar transform’ theory, most simply, confirmation of the integrity of the general micro‐circuitry and plasticity of the cerebellum via eyeblink conditioning could be seen as a sample of global cerebellar health across all functional domains. However, there are obvious caveats to this approach. For example, an alternate viewpoint is that the complete range of cerebellar function entails multiple specialized algorithms across different cerebellar regions.^24^ In this scenario the same underlying circuit implements functionally distinct algorithms subserving different functional modules and tasks (‘multiple functionality’).^16^, ^24^ Correspondingly, it may be that eyeblink conditioning is not tapping into the specific functionality at play in dystonia pathophysiology, a specific algorithm that underwrites the dystonic phenotype. Overall, mechanistically, it remains largely unknown how the cerebellum interplays with other implicated neuroanatomical nodes in the pathophysiology of isolated dystonia. In the future, we are likely to lean toward techniques that evaluate multiple nodes within the network simultaneously in order to gain broader, less one‐dimensional insight. Synergistic and/or compensatory interactions between nodes may define novel mechanisms in dystonia.

We also did not find evidence to support the hypothesis that tremor and other clinical features such as disease severity or medications influence conditioning. Statistically, when controlling for the covariates age and sex we did not replicate findings in a previous study in cervical dystonia in which impaired eyeblink conditioning was linked to those with head tremor.^8^ Similar to dystonia, a network model of the pathophysiology of tremor is proposed in which the olivo‐cerebellar system is thought to be critical. Patients with essential tremor have had both low and normal levels of eyeblink conditioning documented depending on the paradigm used. However, as cerebellar cells show similar responses across paradigms, further studies are needed to establish the fidelity of findings.^25^ Of note, low levels of cerebellar conditioning do not appear to be a marker of any tremor syndrome as eyeblink conditioning appears to be normal in tremor associated with neuropathy.^26^

Limitations of our study are that the numbers within subgroups of dystonia were small. Although we extended previous conclusions by modeling the covariates age and sex and making comparisons to the larger control group, we were underpowered to fully evaluate subgroups. Indeed, our sample size calculations reveal that large studies are required to confidently assess for changes in eyeblink conditioning behavior and most individual studies in the literature are not adequately powered. For example, to detect a 20% difference in the level of conditioning across groups a minimum of 46 participants per group is required. This does bring into question whether eyeblink conditioning is useful to study in heterogeneous disease groups given its inherent variability. Also, in the context of our study, although identical methods and equipment are documented and confirmed among authors, some of the reasons for variability of conditioning response are still likely to reflect non‐biological influences such as differences in experimental technique across investigators.

Of note, a recent publication by Latorre et al studied ‘classical’ neurophysiological markers of dystonia and used these data to discuss whether primary writing tremor is a type of dystonic tremor.^27^ The present analysis leads us to conclude that the sample size was too small to conclude that abnormalities of eyeblink conditioning alone are a general characteristic of dystonia. Sampling a sufficient number of independent neurophysiological tests remains an alternative manner by which to overcome sample size limitations. However, the precise statistics and reliability^28^, ^29^ of such combinations has yet to be determined.

In summary, the variability of eyeblink conditioning responses across individuals prevents us from drawing an overarching conclusion about the eyeblink conditioning in cohorts of isolated dystonia. On average, eyeblink conditioning appears intact and clinical features of dystonia such as tremor may not significantly modulate the level of conditioning. Future studies are required to elucidate exactly how cerebellar involvement interplays with other key neuroanatomical nodes implicated in a dystonic network.

## Author Roles

(1) Research Project: A. Conception, B. Organization, C. Execution; (2) Statistical Analysis: A. Design, B. Execution, C. Review and Critique; (3) Manuscript Preparation: A. Writing of the First Draft, B. Review and Critique.

A.S.: 1A, 1B, 1C, 2A, 2B, 2C, 3A, 3B

L.R.: 1A, 1B, 1C, 2A, 3B

A.L.: 1A, 1B, 1C, 3B

E.A.: 1C, 3B

J.T.: 1C, 3B

I.P.: 1C, 3B

B.S.H.: 1C, 3B

K.B.: 2A, 2B, 3B

K.K.: 1B, 3B

M.J.E.: 1A, 3B

K.P.B.: 1A, 3B

J.C.R.: 1A, 3B

## Full financial disclosures for the previous 12 months

A.S.: Chadburn Clinical Lectureship in Medicine. K.K.: Academy of Medical Sciences Springboard Award (SBF006\1052).

## Supporting information


**Appendix S1.** Supporting Information.

## Data Availability

Data available on request from the authors
